# Systemic Therapy Combined with Locoregional Therapy Improved Survival in Oligometastatic Breast Cancer: A Single-Center Retrospective Cohort Study

**DOI:** 10.1155/2022/7839041

**Published:** 2022-09-20

**Authors:** Yuyu Ma, Fangfang Duan, Yue Zhuang, Chenge Song, Jiajia Huang, Wen Xia, Ruoxi Hong, Qiufan Zheng, Shusen Wang, Yanxia Shi, Fei Xu, Zhongyu Yuan, Xiwen Bi

**Affiliations:** Department of Medical Oncology, Sun Yat-sen University Cancer Center, State Key Laboratory of Oncology in South China, Collaborative Innovation Center for Cancer Medicine, Guangzhou, China

## Abstract

The optimal therapeutic options, adding locoregional therapy (LRT) to systemic therapy (ST) or not, for patients with oligometastatic breast cancer (OMBC) have not been fully elucidated. Hence, we designed a retrospective observational study which enrolled patients with measurable extracranial OMBC having less than 5 metastatic lesions not necessarily in the same organ. We retrospectively reviewed a total of 199 patients diagnosed with extracranial OMBC, including 28 receiving ST followed by LRT (ST to LRT group), 44 receiving LRT followed by ST (LRT to ST group), and 127 receiving ST alone (ST alone group). After a median follow-up of 28.7 months, patients receiving both ST and LRT had a significantly better prognosis than those receiving ST alone: the median progression-free survival (PFS) was 16.3, 14.0, and 9.3 months (*P* < 0.001) and the median overall survival (OS) was 39.8, 70.5, and 26.7 months (*P* < 0.001) in the ST to LRT, LRT to ST, and ST alone groups, respectively. Sequence of ST and LRT had no significant impact on survival among patients receiving both. Further exploratory analysis identified ST plus LRT as an independent predictor for longer PFS. In conclusion, we demonstrated that adding LRT to ST was associated with survival benefits for patients with OMBC, and further prospective studies were warranted.

## 1. Introduction

The prognosis of metastatic breast cancer (MBC) is quite poor [1]. The oligometastatic disease, an intermediate state between localized disease and extensive metastatic disease, presents with limited disease, less invasive behavior, and a better prognosis compared with the common MBC [2]. The precise incidence of oligometastatic breast cancer (OMBC) is unknown, but it is reported represent up to 21.9% of patients with MBC [3]. The 5-year progression-free survival (PFS) of OMBC ranged from 25 to 57% and the 5-year overall survival (OS) ranged from 30 and 79% [4].

With the development of techniques, local treatments are promising for improving the survival of patients. Surgical resection, radiation therapy, thermal ablation therapy, and transcatheter arterial (chemo) ablation all could be used in OMBC. The surgical resection could remove the metastatic lesions completely and relieve the patient's symptoms caused by local compression availably. Radiation therapy is noninvasive which delivers high doses of radiation to small tumor targets and is used to target lesions in the lungs, liver, bone, and adrenals. Thermal therapy also is an important clinical treatment method for some solid tumors with a low rate of complications. Transcatheter arterial (chemo) ablation has been considered as a promising targeted delivery approach for hepatic carcinoma.

Previous studies have suggested that OMBC might benefit from locoregional therapy [5–8]. However, whether such benefit seen in those studies resulted from the efficacy of locoregional treatment itself or selection bias due to the favorable inclusion criteria remained controversial [9]. In addition, the optimal sequence of locoregional and systemic therapy remains to be investigated. Herein, our study was aimed at investigating the impact of locoregional therapy and the optimal sequence of treatment modalities on the prognosis of patients with OMBC.

## 2. Methods

### 2.1. Eligible Patients

**Figure 1:**
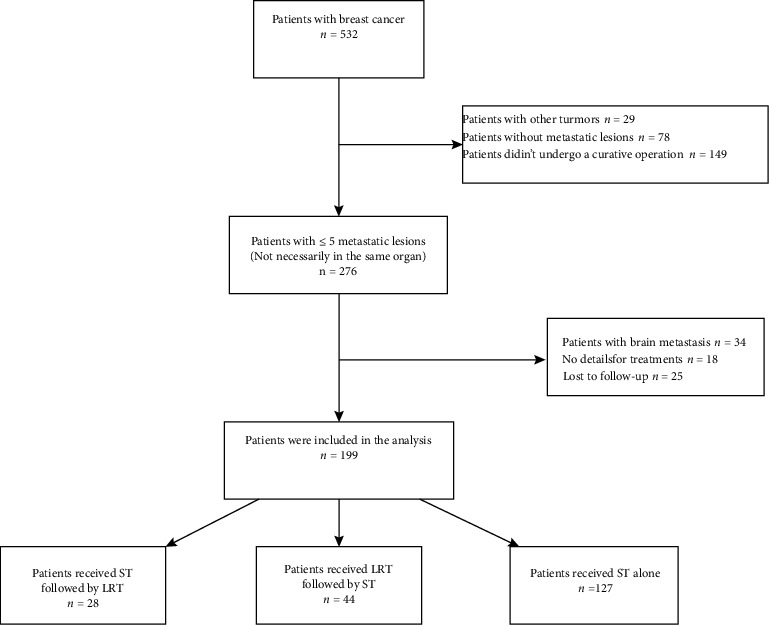
Study flow chart. ST: systemic therapy; LRT: locoregional therapy.

We retrospectively reviewed patients diagnosed with extracranial OMBC between January 2005 and December 2012 at the Sun Yat-sen University Cancer Center (SYSUCC). The definition of OMBC was breast cancer patients with metastatic disease at up to five sites and not necessarily in the same organ. Diagnosis of metastasis was made by pathological examination whenever possible. However, imaging results could be used for diagnosis if rebiopsy is not available. This study was reviewed and approved by the ethics committee of SYSUCC. We performed it following the Declaration of Helsinki. Written informed consent from the patients was waived owing to the retrospective nature of the current study.  Figure 1 presents the flow chart of the study.

Inclusion criteria were as follows: (1) patients who had undergone a curative operation before the diagnosis of OMBC; (2) ≤5 metastatic lesions (not necessarily in the same organ), which were measurable according to Response Evaluation Criteria in Solid Tumors (RECIST) version 1.1; and (3) receiving systemic therapy alone or in combination with locoregional therapy for OMBC. Locoregional therapy included local surgery, radiotherapy, thermal ablation therapy, and transcatheter arterial (chemo) ablation. The sequence of locoregional and systemic therapy was at the physician's discretion.

The exclusion criteria included the following: (1) pleural or pericardial effusion, or ascites at diagnosis of OMBC; (2) locoregional relapse alone in ipsilateral breast; (3) brain metastasis or unresectable lesion in chest wall or skin; and (4) accompanied by other malignancies or life-threatening comorbidities.

### 2.2. Follow-Up and Endpoints

We obtained follow-up information of enrolled patients from the outpatient electronic records of SYSUCC and telephonic interviews. Patients were evaluated every 3 months, including routine physical examination, hematological and laboratory tests, breast and abdominal ultrasonography, computed tomography (CT), or magnetic resonance imaging (MRI). Bone scans were performed if necessary.

The primary endpoint of our study was PFS, which was defined as the time from the first day of diagnosis of OMBC to the date of first disease progression, death due to any cause, or last follow-up. The secondary endpoint was the OS, which was defined as the time from the first day of diagnosis of OMBC to the date of last follow-up or death due to any cause. The disease-free interval (DFI) was defined as the duration from the initial curative surgery to the first detection of oligometastases.

### 2.3. Statistical Analysis

Continuous variables were shown as median values with range. Categorical variables were presented as frequencies with percentages. Comparisons of variables among groups were performed using the chi-square test or Fisher's exact test. The median PFS and OS were estimated using the Kaplan-Meier method, and comparisons among groups were made using the log-rank test. A *P* value of <0.05 was considered significant. Multivariate analysis was carried out using the Cox regression model, and a *P* value of 0.20 was used for covariate entry. Statistical analyses were performed using SPSS (version 22.0, IBM, Armonk, NY, USA). Graphs were created using the GraphPad Prism (version 9.0) and R.

## 3. Results

### 3.1. Demographics and Clinical Characteristics

Between January 2005 and December 2012, a total of 199 patients with OMBC were enrolled in the final analysis, including 28 receiving systemic therapy followed by locoregional therapy (ST to LRT group), 44 receiving locoregional therapy followed by systemic therapy (LRT to ST group), and 127 receiving systemic therapy alone (ST alone group). The demographics and clinical characteristics of the patients are summarized in Table 1. Patients receiving ST alone had significantly more metastatic lesions than those treated in the ST to LRT group and the LRT to ST group (4-5 sites: 66.9% vs. 25.0% and 29.5%, respectively, *P* < 0.001). There was a significantly higher proportion of lung metastasis in patients receiving ST alone than in other groups (35.4% vs. 10.7% and 9.1%, respectively, *P* < 0.001). Other remaining characteristics were not significantly different among the three groups.

### 3.2. Treatments and Response Evaluation

Treatments for OMBC are summarized in Table 2. Systemic therapy included chemotherapy, endocrine therapy, and anti-HER2 therapy. Locoregional therapy included surgical resection, radiation therapy, thermal ablation therapy, and transcatheter arterial (chemo) ablation. Most patients received surgical resection or radiation therapy as locoregional therapy. The response evaluation of systemic therapy and locoregional therapy was performed according to RECIST guidelines version 1.1. Only one patient was not evaluated during the entire assessment. For 28 assessable patients in the ST to LRT group, 15 (53.6%) showed a complete response (CR), and 9 (32.1%) showed a partial response (PR). For the 44 evaluated patients treated in the LRT to ST group, CR and PR were observed in 28 (63.6%) and 10 (22.7%), respectively. As for the ST alone group, there were only 12 (9.4%) patients that had a CR, and 15 (11.8%) patients progressed. The overall response rate (ORR) among the ST to LRT group, LRT to ST group, and ST alone group was significantly different (85.7% vs. 86.4% vs. 65.4%, respectively, *P* = 0.007) (Table 3).

### 3.3. Survival Outcome

**Figure 2:**
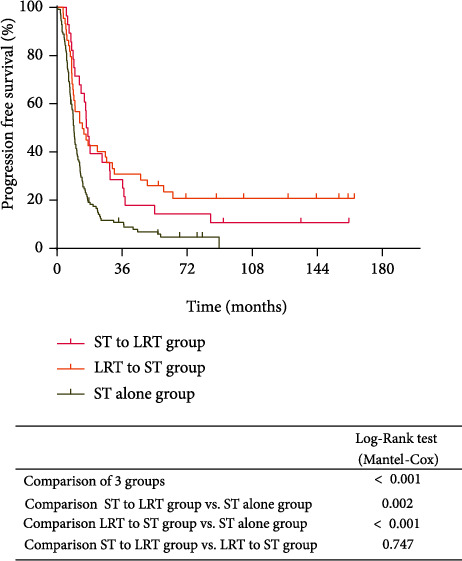
Kaplan-Meier survival for progression-free survival (PFS) according to the treatment approach. Patients who received both systemic and locoregional therapies had significantly longer PFS than those who received systemic therapy alone. The median PFS was 16.3, 14.0, and 9.3 months in the ST to LRT, LRT to ST, and ST alone groups, respectively. ST to LRT: systemic therapy followed by locoregional therapy; LRT to ST: locoregional therapy followed by systemic therapy; ST: systemic therapy.

**Figure 3:**
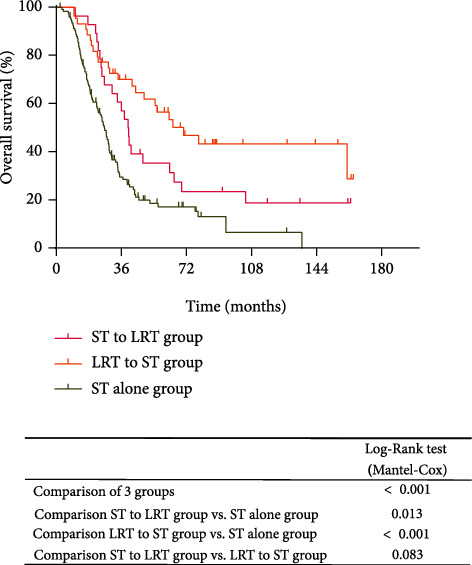
Kaplan-Meier survival for overall survival (OS) according to the treatment approach. Patients who received both systemic and locoregional therapies had significantly longer OS than those who received systemic therapy alone. The median OS was 39.8, 70.5, and 26.7 months in the ST to LRT, LRT to ST, and ST alone groups, respectively. ST to LRT: systemic therapy followed by locoregional therapy; LRT to ST: locoregional therapy followed by systemic therapy; ST: systemic therapy.

The median follow-up was 28.7 months (range: 2.2-164.5months). There was a significant difference in median PFS among the ST to LRT, LRT to ST, and ST alone groups (16.3 vs. 14.0 vs. 9.3 months, respectively, *P* < 0.001; Figure 2). Patients in the ST to LRT group and the LRT to ST group had significantly better PFS compared with those in the ST alone group (*P* = 0.002 and *P* < 0.001, respectively). However, there was no significant difference in terms of PFS between ST to LRT and LRT to ST groups (*P* = 0.747). Similarly, there was a significant difference among the three groups for OS (*P* < 0.001; Figure 3). The median OS was 39.8, 70.5, and 26.7 months in the ST to LRT, LRT to ST, and ST alone groups, respectively. In addition, patients treated with the ST to LRT and LRT to ST showed longer OS than those receiving ST alone (*P* = 0.013 and *P* < 0.001, respectively), whereas there was no significant difference between ST to LRT and LRT to ST groups (*P* = 0.083).

### 3.4. Exploratory Analysis

**Figure 4:**
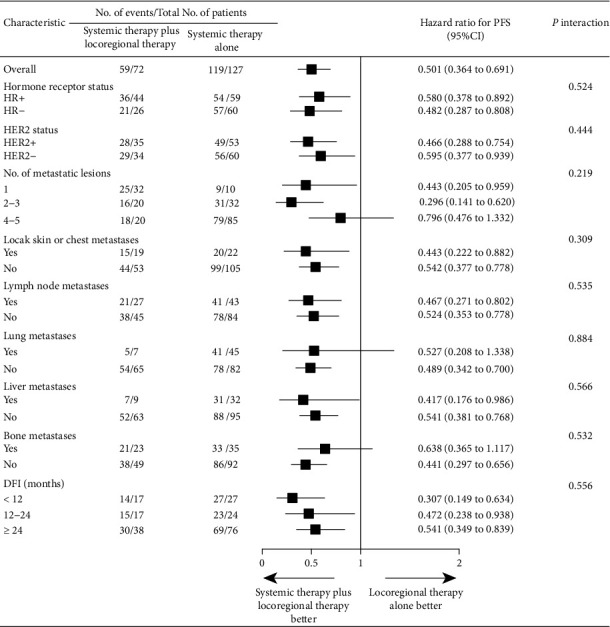
Subgroup analysis of progression-free survival (PFS). HR: hormone receptor; HER2: human epidermal growth factor receptor 2; DFI: disease-free interval.

According to the multivariate analysis, we revealed that ST plus LRT, DFI ≥ 24 months, and hormone receptor- (HR-) positive tumors served as independent prognostic factors for longer PFS (Table 4). Considering the small sample sizes of the ST to LRT and LRT to ST groups, we then divided all available patients into the ST plus LRT group and the ST alone group. Further exploratory analysis revealed that most patients in the systemic therapy plus locoregional therapy group could benefit, except for patients with 4-5 metastatic lesions (HR = 0.796, 95% CI 0.476-1.332, *P* = 0.219) or with the presence of lung metastases (HR = 0.527, 95% CI 0.208-1.338, *P* = 0.884) or with bone metastases (HR = 0.638, 95% CI 0.365-1.117, *P* = 0.532) (Figure 4).

## 4. Discussion

The conception of oligometastases was firstly proposed by Hellman and Weichselbaum [10]. Subsequently, abundant research pointed out that locoregional therapy could significantly improve the survival of patients with OMBC.

Our study was aimed at investigating the impact of locoregional therapy and the optimal sequence of treatment modalities on the prognosis of patients with OMBC. We excluded patients who met the aforementioned exclusion criteria. Firstly, patients with pleural or pericardial effusion or ascites are generally unable to receive local irradiation or resection. Secondly, locoregional relapse alone in the ipsilateral breast is generally considered potentially curable, and most of those patients will receive systemic therapy followed by surgical resection and irradiation (if possible) in our hospital [11]. Thirdly, patients with brain metastasis are insensitive to systemic therapy, and those with symptomatic brain metastasis almost always require locoregional treatment to mitigate symptoms, and patients with unresectable lesion in the chest wall or skin are generally treated with systemic therapy in combination with local irradiation. Moreover, patients accompanied by other malignancy or life-threatening comorbidities are also largely not treated locally. Patients who met the first or fourth exclusion criterion do not receive locoregional therapy mostly, and those patients who met the second or third exclusion criterion almost always receive locoregional therapy. Therefore, we excluded the above patient to reduce selection bias.

Our data also indicated that OMBC patients in the ST to LRT group and the LRT to ST group had better PFS and OS than those in the ST alone group, which was consistent with most previous studies. Therefore, we believed that systemic therapy combined with locoregional therapy could significantly improve the survival of OMBC. To our knowledge, our study is the largest single-center real-world analysis of OMBC to date.

Numerous retrospective studies have exhibited that locoregional therapy could prolong the PFS of OMBC. In the study conducted by Lan et al., OMBC was defined as the number of metastatic lesions that was no more than three, and metastatic diseases were limited to a single organ. Finally, 20 patients received systemic treatment and surgical resection, 10 patients received systemic treatment and local treatment other than resection, and 20 patients received systemic treatment alone. The median PFS was 49.6 months, 13.8 months, and 6.9 months, respectively, and the 2-year PFS rate was 65%, 30%, and 20%, respectively [5]. Concerning patients with HR-positive and HER2-negative OMBC, Cha et al. found that the median PFS was significantly longer in patients with local treatment than in patients without local treatment (30.0 vs. 18.0 months, *P* = 0.049) [12]. Lemoine et al. pointed out that the median PFS of OMBC treated with stereotactic body radiation therapy appeared longer with low toxicity. The local control rate was 100% at three years [13]. Research carried out by Wijetunga et al. also reported that long-term systemic disease control and survival could be achieved with stereotactic ablative body radiotherapy (SABR) for OMBC [14].

Besides, some phase II clinical trials also demonstrated that OMBC patients receiving locoregional therapy had better survival than those who did not. In the preliminary report of the SABR-COMET trial, 99 eligible patients were randomized to the control group and SABR group in a 1 : 2 ratio; after a median follow-up of 25 months in the control group and 26 months in the SABR group, they showed that the median PFS was 6 months and 12 months, respectively (*P* = 0.0012) [7]. In the second report, with a longer median follow-up of 51 months, they again revealed that the median PFS was better in the SABR group than in the control group (11.6 vs. 5.4 months, *P* = 0.001) [8]. Likewise, Trovo et al. also have revealed that patients with OMBC treated with radical radiotherapy to all metastatic sites might achieve long PFS. Of note, 48 patients in this study received locoregional therapy plus systemic therapy [15]. Hence, we believed that OMBC could benefit from locoregional therapy plus systemic therapy.

All the above studies suggested the potential survival benefit of locoregional therapy for OMBC, but it was not clear which locoregional therapy was optimal. The innovation of our study was that we also explored whether the sequence of systemic therapy and locoregional therapy could affect survival. We found that there was no significant difference between the ST to LRT group and the LRT to ST group, similar to the study of Lan et al., which noted that among patients treated with surgical resection, there was no difference in PFS between patients having resection first and those having systemic therapy first (*P* = 0.807) [5]. However, Cha et al. argued that systemic therapy should be made after or at the same time as local therapy to control the residual microlesions [12]. The conventional perspective proposed that more aggressive tumors should receive systemic therapy first to eliminate drug-sensitive cells, followed by locoregional therapy to eradicate drug-resistant cells. According to our data, we consumed that radically locoregional therapy before or after systemic therapy might not influence the survival of OMBC.

However, it should not be ignored that there are several factors that affect the sequence of systemic therapy and locoregional therapy, including the size and location of metastatic lesions and symptom of patients. Generally, the sequence of local and systemic therapies was at the physician's personal discretion. For example, if metastases are asymptomatic or difficult to be resected, we prefer to choose systemic treatment first. On the contrary, if the local metastases cause severe symptoms that affect the patient's quality of life or threaten the patient's life, we tend to choose locoregional therapy first. Certainly, large prospectively clinical studies were needed to address this issue.

Moreover, we also found that locoregional therapy combined with systemic therapy significantly improved OS of OMBC. The preliminary report from SABR-COMET also suggested that patients receiving SABR achieved an improvement of 13 months in OS [7]. A real-world study including 3447 patients diagnosed with OMBC conducted by Steenbruggen et al. also indicated that locoregional therapy of metastases is associated with better OS [16]. However, Cha et al. failed to find a significant difference in OS between these two groups [12]. Therefore, new prospective or multicenter studies are warranted to find whether local treatment would affect the OS of OMBC.

The precise definition of OMBC has not been elucidated. We enroll the patients with less than 5 measurable metastatic lesions not necessarily in the same organ. First, many patients who develop metastases usually involved multiple organs not just single. Second, limiting the number of metastases to less than 5 may help us to enroll sufficient patients and enhance the facility of the study further. Third, it is advantageous for us to explore whether the number of metastases affects the survival of OMBC in the subsequent subgroup analysis. Moreover, most of the previous studies used less than 5 lesions as the definition of “oligo” [17–20].

The exploratory analysis of our study discovered that patients with 4-5 metastatic lesions could not benefit from locoregional treatment. Based on this interesting finding, we believed that a precise definition of OMBC was crucial, whereas there was no clear and standard definition of OMBC to date. Some literature defined oligometastases as the number of metastases of no more than 3, while other studies defined it as the number less than 5, even less than 8 metastatic lesions [7, 8, 15, 16, 21]. Some studies suggested that the definition of oligometastases not only focused on the number of metastatic lesions but also was dedicated to whether locoregional treatment could be implemented within an acceptable safe range [22]. Some literature specifically limited the diameter or the volume size of metastatic lesions [23, 24]. As for the optimal threshold for OMBC, a real-world study uncovered that the ten-year OS rates estimated for patients with no more than 3 versus more than 3 metastases were 14.9% and 3.4% (*P* < 0.001), respectively, and subsequently revealed that 3 limited metastases appeared to the optimal cutoff to define OMBC [16]. Hence, we believed that the number of metastatic lesions of no more than 3 might be appropriate to select the most optimal population to deliver locoregional treatment.

In addition, our exploratory analysis also found that patients with bone or lung metastasis might not benefit from locoregional therapy, which was paradoxical to previous studies [25–27]. We listed two possible reasons for this finding. First, depending on our data, the number of metastatic lesions in patients with bone metastases and lung metastases was mostly 4-5 (67.2% and 82.7%, respectively), which might hardly benefit from local treatment. Second, a majority of patients with bone metastases and lung metastases received systemic therapy alone (60.3% and 86.5%, respectively), and fewer received locoregional therapy plus systemic therapy.

Moreover, we found that the addition of locoregional therapy, DFI ≥ 24 months, and primary tumor lesions being HR-positive were associated with better survival of patients with OMBC, which were in accordance with previous studies and meaningful to select ideal subpopulations to receive locoregional treatment [28–30]. Although the latest results of the prospective, phase IIR/III NRG-BR002 trial presented that the comparison of median PFS between standard systemic plus local therapy and standard systemic therapy groups failed to reach predetermined statistical difference (30.0 vs. 19.5 months, respectively, *P* = 0.36), considering previous positive results, the NRG-BR002 trial thought that it was warranted to screen potential subpopulations for benefiting from additional local care to standard systemic therapy. There are numerous prospective studies (OLIGOMA trial, NCT04413409, NCT04646564) to evaluate the efficacy of locoregional therapy for OMBC; we look forward to their results [31].

There were several limitations of the current study to be noted. First, the follow-up was relatively short; we will continue to monitor patients and pay attention to their long-term follow-up results. Second, this was a retrospective analysis from a single center; selective biases might be inevitable, which is a common problem for retrospective studies. Therefore, we hoped to validate our results in multicenter, prospective cohorts in the future. Third, there was heterogeneity in the modality of locoregional treatment. Thus, the result of this study should be interpreted with caution.

## 5. Conclusion

In summary, our study confirmed that adding locoregional therapy to systemic therapy significantly improved the survival of patients with OMBC. The sequence of systemic therapy and locoregional therapy had no impact on prognosis. Future prospective randomized studies are warranted to verify the appropriate role of locoregional therapy, as well as the optimal sequence of locoregional therapy and systemic therapy.

## Figures and Tables

**Table 1:** Demographics and clinical characteristics.

Variable	ST to LRT group	LRT to ST group	ST alone group	*P* value
Age				0.131
Median(range)	43.0 (22-57)	46.5 (28-83)	45.0 (23-70)	
ECOG.ps [*n* (%)]				0.775
0	15 (53.6)	28 (63.6)	69 (54.3)	
1	13 (46.4)	16 (36.4)	56 (44.1)	
2	0 (0.0)	0 (0.0)	2 (1.6)	
Stage at initial diagnosis (UICC 7^th^) [*n* (%)]				0.553
I	0 (0.0)	2 (4.5)	7 (5.5)	
II	14 (50.0)	23 (52.3)	49 (38.6)	
III	12 (42.9)	14 (31.8)	59 (46.5)	
Unknown	2 (7.1)	5 (11.4)	12 (9.4)	
Pathologic type [*n* (%)]				0.185
IDC	23 (82.1)	37 (84.1)	114 (89.8)	
ILC	2 (7.1)	2 (4.5)	1 (0.8)	
Unknown or others	3 (10.7)	5 (11.4)	12 (9.4)	
Immunohistochemical subtype [*n* (%)]				0.058
HR+HER2-	7 (25.0)	18 (40.9)	31 (24.4)	
HR+HER2+	11 (39.3)	7 (15.9)	23 (18.1)	
HR-HER2+	6 (21.4)	11 (25.0)	30 (23.6)	
TNBC	4 (14.3)	5 (11.4)	29 (22.8)	
Numbers of metastatic lesions [*n* (%)]				<0.001
1	11 (39.3)	21 (47.7)	10 (7.9)	
2-3	10 (35.7)	10 (22.7)	32 (25.2)	
4-5	7 (25.0)	13 (29.5)	85 (66.9)	
Metastatic site [*n* (%)]				
Local skin or chest	6 (21.4)	13 (29.5)	22 (17.3)	0.223
Lymph node	11 (39.3)	16 (36.4)	43 (33.9)	0.847
Lung	3 (10.7)	4 (9.1)	45 (35.4)	<0.001
Liver	4 (14.3)	5 (11.4)	32 (25.2)	0.099
Bone	12 (42.9)	11 (25.0)	35 (27.6)	0.215
Others	0 (0.0)	3 (6.8)	6 (4.7)	0.463
Previous neoadjuvant/adjuvant chemotherapy [*n* (%)]				0.380
Anthracycline alone	10 (35.7)	21 (47.7)	53 (41.7)	
Taxane alone	0 (0.0)	2 (4.5)	2 (1.6)	
Anthracycline+taxane	10 (35.7)	11 (25.0)	50 (39.4)	
None or unknown	8 (28.6)	10 (22.7)	22 (17.3)	
Previous adjuvant endocrine therapy [*n* (%)]				0.232
TAM/TOR	13 (46.4)	21 (47.7)	40 (31.5)	
AI	1 (3.6)	3 (6.8)	4 (3.1)	
None	14 (50.0)	19 (43.2)	79 (62.2)	
Unknown or others	0 (0.0)	1 (2.3)	4 (3.1)	
Previous adjuvant anti-HER2 therapy [*n* (%)]				0.728
Trastuzumab	0 (0.0)	0 (0.0)	3 (2.4)	
None or unknown	28 (100)	44 (100)	124 (97.6)	

Abbreviations: ST to LRT: systemic therapy followed by locoregional therapy; LRT to ST: locoregional therapy followed by systemic therapy; ST: systemic therapy; ECOG.ps: Eastern Cooperative Oncology Group performance status; UICC 7^th^: Union for International Cancer Control, the seventh edition; IDC: invasive ductal carcinoma; ILC: invasive lobular carcinoma; HR: hormone receptor; HER2: human epidermal growth factor receptor 2; TNBC: triple negative breast cancer; TAM: tamoxifen; TOR: toremifene; AI: aromatase inhibitor.

**Table 2:** Treatment after diagnosis of oligometastases.

Treatment	ST to LRT group (*n* = 28)	LRT to ST group (*n* = 44)	ST alone group (*n* = 127)
Systemic therapy [*n* (%)]			
Chemotherapy	26 (92.9)	32 (72.7)	124 (97.6)
Endocrine therapy	12 (42.9)	23 (52.3)	41 (32.3)
Anti-HER2 therapy	5 (17.9)	6 (13.6)	19 (15.0)
Locoregional therapy [*n* (%)]			
Surgery	3 (10.7)	30 (68.2)	
Radiotherapy	22 (78.6)	19 (43.2)	
Thermal ablation therapy	3 (10.7)	2 (4.5)	
Transcatheter arterial (chemo) ablation	0 (0.0)	1 (2.3)	

Abbreviations: ST to LRT: systemic therapy followed by locoregional therapy; LRT to ST: locoregional therapy followed by systemic therapy; ST: systemic therapy.

**Table 3:** Responses after treatment.

Viable	ST to LRT group (*n* = 28)	LRT to ST group (*n* = 44)	ST alone (*n* = 127)	*P* value
Best response				<0.001
CR	15 (53.6)	28 (63.6)	12 (9.4)	
PR	9 (32.1)	10 (22.7)	71 (55.9)	
SD	4 (14.3)	6 (13.6)	28 (22.0)	
PD	0 (0.0)	0 (0.0)	15 (11.8)	
Not evaluated	0 (0.0)	0 (0.0)	1 (0.8)	
ORR	24 (85.7)	38 (86.4)	83 (65.4)	0.007

Abbreviations: ST to LRT: systemic therapy followed by locoregional therapy; LRT to ST: locoregional therapy followed by systemic therapy; ST: systemic therapy; CR: complete response; PR: partial response; SD: stable disease; PD: progression disease; ORR: overall response rate.

**Table 4:** Univariate analysis and multivariate analysis of progression-free survival.

Variable	Univariate analysis			Multivariate analysis		
	Hazard ratio	95% CI	*P* value	Hazard ratio	95% CI	*P* value
Hormone receptor status						
HR+	1			1		
HR-	1.719	1.264-2.338	0.001	1.636	1.190-2.249	0.002
HER2 status						
HER2+	1					
HER2-	1.122	0.824-1.528	0.464			
No. of metastatic lesions						
1	1			1		
2-3	1.457	0.936-2.268	0.095	1.192	0.737-1.929	0.474
4-5	1.656	1.116-2.456	0.012	1.140	0.697-1.864	0.602
Chest or skin metastasis						
No	1			1		
Yes	0.780	0.537-1.131	0.190	0.909	0.613-1.348	0.635
Lymph node metastasis						
No	1					
Yes	0.857	0.629-1.168	0.329			
Lung metastasis						
No	1					
Yes	1.212	0.865-1.697	0.263			
Liver metastasis						
No	1			1		
Yes	1.294	0.904-1.853	0.159	1.035	0.712-1.506	0.856
Bone metastasis						
No	1					
Yes	1.068	0.775-1.472	0.686			
DFI (months)						
<12	1			1		
12-24	0.841	0.540-1.307	0.441	0.785	0.498-1.237	0.296
≥24	0.695	0.483-1.002	0.051	0.626	0.427-0.918	0.017
Treatment approach						
ST alone	1			1		
ST to LRT	0.527	0.341-0.814	0.004	0.518	0.319-0.840	0.008
LRT to ST	0.484	0.328-0.715	<0.001	0.491	0.310-0.778	0.002

Abbreviations: CI: confidence interval; HR: hormone receptor; HER2: human epidermal growth factor receptor 2; DFI: disease-free interval; ST to LRT: systemic therapy followed by locoregional therapy; LRT to ST: locoregional therapy followed by systemic therapy; ST: systemic therapy.

## Data Availability

The data analyzed in this study are available from one of the corresponding authors (Xiwen Bi, E-mail: bixw@sysucc.org.cn) on reasonable requests.
